# NF-κB functions as a molecular link between tumor cells and Th1/Tc1 T cells in the tumor microenvironment to exert radiation-mediated tumor suppression

**DOI:** 10.18632/oncotarget.8246

**Published:** 2016-03-21

**Authors:** Priscilla S. Simon, Kankana Bardhan, May R. Chen, Amy V. Paschall, Chunwan Lu, Roni J. Bollag, Feng-Chong Kong, JianYue Jin, Feng-Ming Kong, Jennifer L. Waller, Raphael E. Pollock, Kebin Liu

**Affiliations:** ^1^ Department of Biochemistry and Molecular Biology, Medical College of Georgia, Augusta, GA, USA; ^2^ Radiation Oncology, Medical College of Georgia, Augusta, GA, USA; ^3^ Biostatistics and Epidemiology, Medical College of Georgia, Augusta, GA, USA; ^4^ Cancer Center, Georgia Regents University, Augusta, GA, USA; ^5^ Ohio State University Medical Center, Columbia, OH, USA; ^6^ Charlie Norwood VA Medical Center, Augusta, GA, USA

**Keywords:** NF-κB, radiation, TNFα, cytotoxic T lymphocyte, Smac mimetic

## Abstract

Radiation modulates both tumor cells and immune cells in the tumor microenvironment to exert its anti-tumor activity; however, the molecular connection between tumor cells and immune cells that mediates radiation-exerted tumor suppression activity in the tumor microenvironment is largely unknown. We report here that radiation induces rapid activation of the p65/p50 and p50/p50 NF-κB complexes in human soft tissue sarcoma (STS) cells. Radiation-activated p65/p50 and p50/p50 bind to the *TNFα* promoter to activate its transcription in STS cells. Radiation-induced TNFα induces tumor cell death in an autocrine manner. A sublethal dose of Smac mimetic BV6 induces cIAP1 and cIAP2 degradation to increase tumor cell sensitivity to radiation-induced cell death *in vitro* and to enhance radiation-mediated suppression of STS xenografts *in vivo*. Inhibition of caspases, RIP1, or RIP3 blocks radiation/TNFα-induced cell death, whereas inhibition of RIP1 blocks TNFα-induced caspase activation, suggesting that caspases and RIP1 act sequentially to mediate the non-compensatory cell death pathways. Furthermore, we determined in a syngeneic sarcoma mouse model that radiation up-regulates IRF3, IFNβ, and the T cell chemokines CCL2 and CCL5 in the tumor microenvironment, which are associated with activation and increased infiltration of Th1/Tc1 T cells in the tumor microenvironment. Moreover, tumor-infiltrating T cells are in their active form since both the perforin and FasL pathways are activated in irradiated tumor tissues. Consequently, combined BV6 and radiation completely suppressed tumor growth *in vivo*. Therefore, radiation-induced NF-κB functions as a molecular link between tumor cells and immune cells in the tumor microenvironment for radiation-mediated tumor suppression.

## INTRODUCTION

Radiotherapy is the mainstay local treatment for many types of solid cancers, and about two thirds of cancer patients require radiation at least once during the course of their disease treatment. However, local failure is frequent due to tumor recurrence, and survival rate after radiotherapy for many human cancers is not high [1-3]. Therefore, any improvement in radiotherapy efficacy will benefit a large number of human patients [3]. A direct consequence of radiation is DNA damage and DNA damage-mediated cell death. Recent studies, however, indicate that multiple biological effects play a key role in radiation-induced tumor suppression. In addition, radiation may increase various signaling transduction pathways in the tumor cells to increase the tumor cell sensitivity to chemotherapeutic agents [3, 4]. Interestingly, radiation may also alter the inflammatory tumor microenvironment through stimulation of host immune cells to suppress tumor growth and progression [4-9]. However, the molecular mechanisms underlying radiation-mediated biological and immunological effects are still elusive.

Low levels of constitutive NF-κB activity are present in many types of human cancers, and NF-κB activation often promotes tumor growth and progression [10-13]. This provides a strong rationale for anticancer strategies that inhibit NF-κB signaling [14, 15]. In fact, several hundred NF-κB inhibitors have been developed [16]. However, compelling experimental data have also shown that NF-κB may harbor dual functionality by acting to promote apoptosis and senescence in multiple types of cells, including cancer cells [17-34]. In addition to its essential function in tumor cells, the NF-κB pathway also exerts an essential function in the regulation of the immune response [35-37]. Particularly, NF-κB is known to be essential for T cell differentiation and function [35, 38]. T cell-based cancer immunotherapy has shown durable efficacy in human cancer patients [39] and has been approved for treatment of human cancers. Therefore, blocking NF-κB activation might impair T cell activation and function in cancer patients, and its side effects on T cells in the tumor microenvironment should be considered with great care. The molecular mechanisms underlying these contrasting functions of NF-κB in cell death and proliferation are currently unknown. In this study, we determine that radiation-activated NF-κB functions as a molecular link between tumor cells and immune cells to exert radiation-induced tumor suppression.

## RESULTS

### Smac mimetic BV6 is effective in sensitization of human tumor cells to radiation-induced cell death

**Figure 1:**
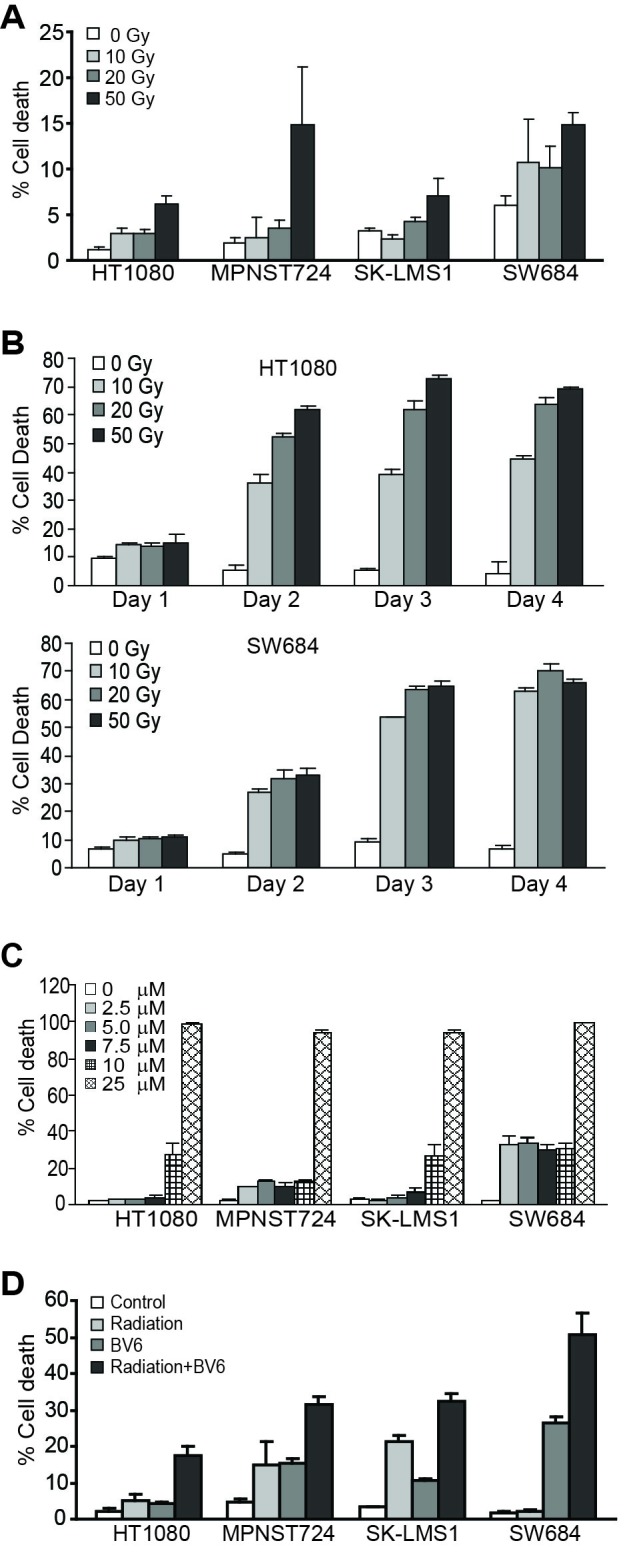
BV6 increases the sensitivity of human soft tissue sarcoma cells to radiation **A.** Tumor cells were irradiated at the indicated doses and cultured for 24 hours. Cells were stained with PI and analyzed by flow cytometry. Percent cell death is expressed as % PI^+^ cells. Column: mean; Bar: SD. **B.** Tumor cells were irradiated with γ-ray at the indicated doses and cultured for 1, 2, 3 and 4 days. Cells were then analyzed as in A. **C.** Tumor cells were cultured in the presence of BV6 at the indicated doses for 24 hours. Cell death was determined as in A. **D.** Tumor cells were either untreated or irradiated (50 Gy) and then cultured in the absence or presence of BV6 (5 μM) for 24h. Cell death was determined as in A.

Human soft tissue sarcoma cell lines HT1080, MPNST724, SK-LMS1, and SW684 were irradiated and cultured for 24 hours. Analysis of cell death indicates that all four cell lines exhibited limited responses to radiation in a one day assay (Figure 1A). Further analysis with two of the tumor cell lines indicate that these tumor cells are sensitive to a dose of 10 Gy or higher after two or more days post radiation *in vitro* (Figure 1B). To test whether inhibition of IAPs increase STS cell sensitivity to radiation [21, 40-44], cells were cultured in the presence of BV6. Analysis of cell death revealed that BV6 induces STS cell death in a dose-dependent manner (Figure 1C). To test the efficacy of BV6 as a sensitizer of radiation-induced cell death, human sarcoma cells were either untreated or irradiated, followed by culture in the absence or presence of a sublethal dose of BV6. A sublethal dose of BV6 significantly increased the sensitivity of all four human sarcoma cell lines to radiation-induced cell death (Figure 1D).

**Figure 2:**
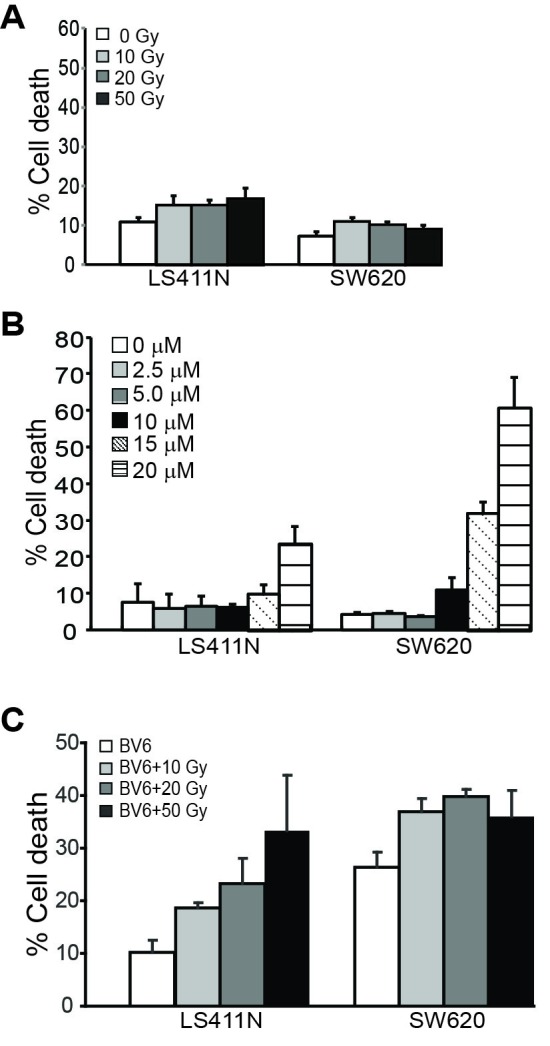
BV6 increases the sensitivity of human colon carcinoma cells to radiation-induced cell death **A.**. Tumor cells were irradiated at the indicated doses and cultured for 24 hours. Cells were stained with PI and analyzed by flow cytometry. Percent cell death was expressed as % PI^+^ cells. Column: mean; Bar: SD. **B.** Tumor cells were cultured in the presence of BV6 at the indicated doses for 24 hours. Cell death was determined as in A. **C.** Tumor cells were irradiated at the indicated dose and then cultured in the presence of BV6 (5 μM) for 24 hours. Cell death was determined as in A.

To determine whether this observation can be extended to other types of human cancer cells, we also examined the effects of radiation and BV6 on human colon carcinoma cells. The human colon carcinoma cell lines SW620 and LS411N are relatively resistant to radiation in a one day assay (Figure 2A). BV6 also exhibits cytotoxicity to these two human colon carcinoma cell lines (Figure 2B). Consistent with observations in human sarcoma cell lines, a sublethal dose of BV6 increased the sensitivity of both SW620 and LS411N cell lines to radiation-induced cell death (Figure 2C).

### cIAP1 protein level indicates poor prognosis of human CRC patients

**Figure 3:**
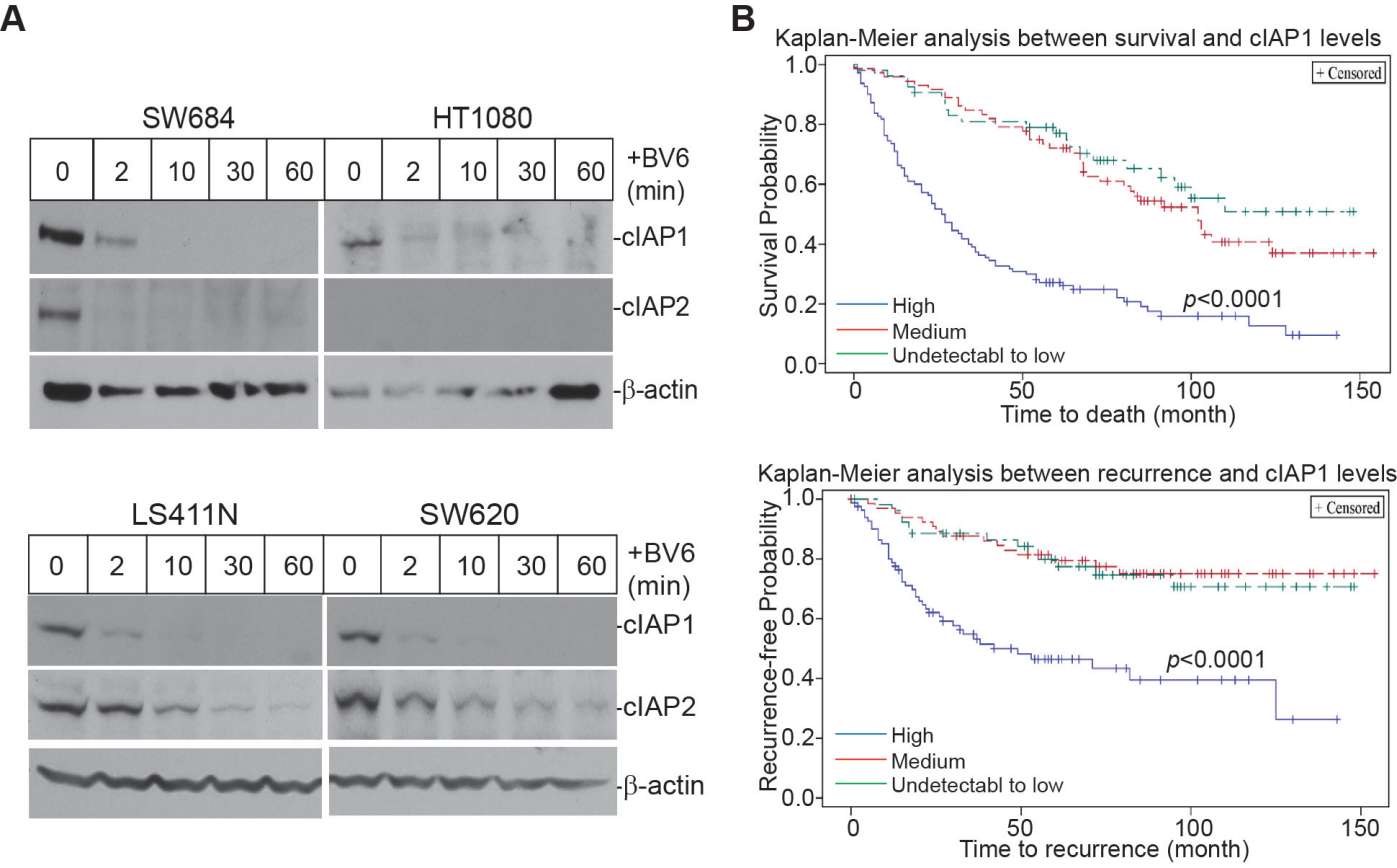
cIAP1 protein level is correlated with shorter survival time and earlier recurrence in human colorectal cancer patients **A.** Human sarcoma (top panel) and colon carcinoma (bottom panel) cells were treated with BV6 (5 μM) for the indicated time and analyzed by Western blotting analysis for cIAP1 and cIAP2 levels. β-actin was used as a normalization control. **B.** TMA slides containing human colorectal cancer specimens (N=235) were stained for cIAP1 protein level. The stained specimens were then statistically analyzed for correlations between cIAP1 protein levels and patient survival time (top panel) or cancer recurrence time (bottom panel). Each variable is indicated by colored lines in the plot.

BV6 is a Smac mimetic that induces IAPs degradation [21, 43, 44]. BV6 treatment resulted in rapid degradation of cIAP1 and cIAP2 in human STS and colon carcinoma cells (Figure 3). Next, we made use of a human colon cancer tissue microarray and stained for cIAP1 proteins. Kaplan-Meier analysis of the 235 human colorectal cancer specimens revealed that the cIAP1 protein level is inversely correlated with disease-specific survival and positively correlated with cancer recurrence (Figure 3B). Patients with high cIAP1 protein levels had a significantly lower survival time as compared to patients with medium to low or undetectable cIAP1 protein levels in the tumor cells. Furthermore, patients with high cIAP1 protein levels in the tumor cells also exhibited a significantly higher recurrence rate as compared to patients with medium to low and undetectable cIAP1 protein levels in the tumor cells (Figure 3B).

### BV6 activates the non-canonical but not the canonical NF-κB pathway

**Figure 4:**
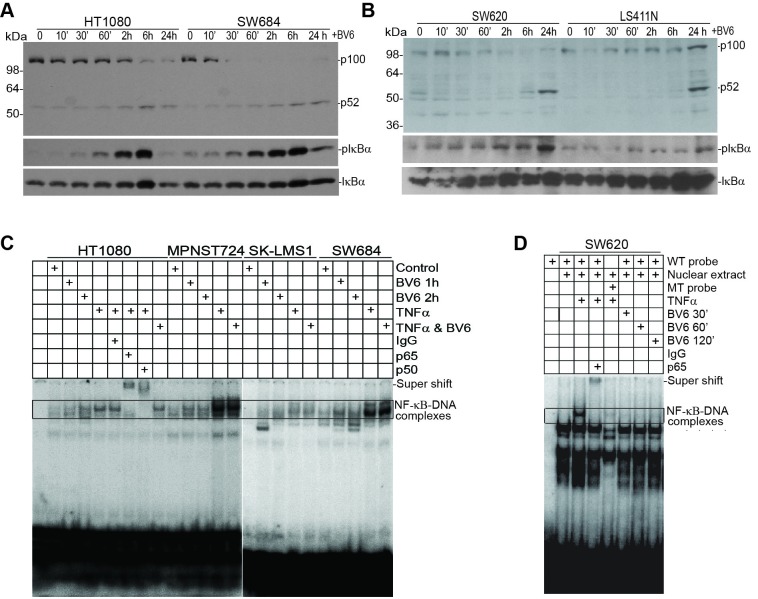
BV6 activates the alternate but not the canonical NF-κB **A.** The indicated human sarcoma cells were treated with BV6 (5 μM) for the indicated time. Cells were then analyzed by Western blotting analysis for p100/p52, pIκBα, and IκBα. **B.** The indicated human colon carcinoma cells were treated with BV6 (5 μM) for the indicated time and then analyzed for p100/p52, pIκBα, and IκBα as in A. **C.** The indicated human sarcoma cells were treated with BV6 (5μM, 1 and 2h), TNFα (100 U/ml, 1h), or both as indicated. Nuclear extracts were prepared from the tumor cells and analyzed for canonical NF-κB activity using EMSA with NF-κB consensus sequence-containing DNA probes (Santa Cruz Biotech). Anti-p65 and anti-p50 antibodies were used to identify the canonical NF-κB-DNA complexes. **D.** SW620 cells were treated with BV6 (5 μM, for 0.5, 1, and 2h) and TNFα (1 h) and analyzed for NF-κB activation as in C.

**Figure 5:**
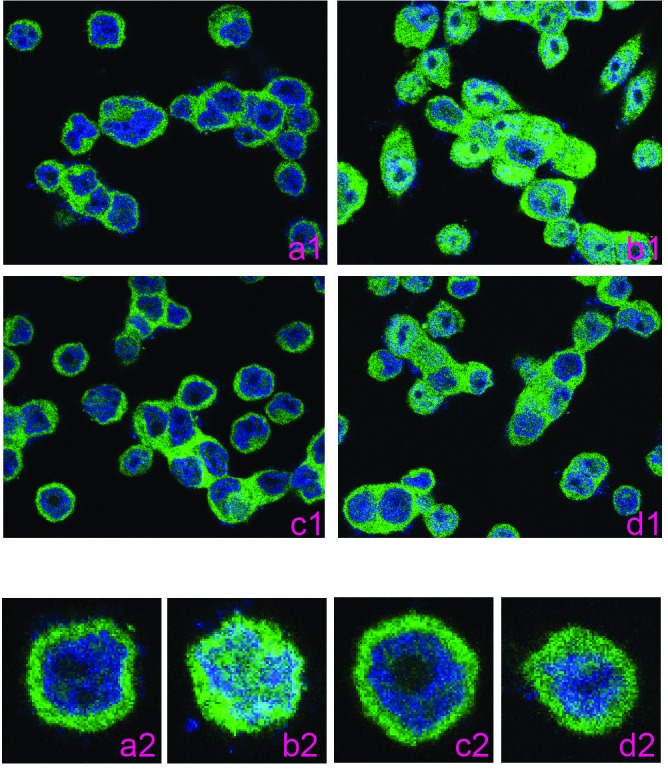
BV6 does not induce NF-κB nuclear translocation SW620 cells were either untreated (a), treated with TNFα (b, 100 U/ml), BV6 (c, 5 μM) or both TNFα and BV6 (d) for 60 min. Cells were fixed, permeabilized, and stained with p65-specific antibody, followed by fluorescent dye-conjugated 2^nd^ antibody. The images were obtained with a confocal microscope. a1-d1 are low amplification images and a2-d2 are high amplification images.

cIAP1 and cIAP2 are also E3 ligases that mediate NF-κB activation [19, 21, 45]. BV6 induced rapid IκBα phosphorylation in human sarcoma cells. Time-dependent p100 processing to p52 was also observed in both sarcoma cell lines (Figure 4A). Similar patterns were also observed in the human colon carcinoma LS411N and SW620 cell lines (Figure 4B). As expected, the positive control TNFα induced activation of the canonical NF-κB as both the p65 and p50 subunits are bound to the DNA probe (Figure 4C). However, BV6 treatment did not induce detectable p65 or p50 binding to the DNA (Figure 4C). Similar outcomes were observed in the human colon carcinoma cells (Figure 4D). A complimentary approach was used to validate the EMSA results. SW620 cells were untreated or treated with TNFα, BV6, or both TNFα and BV6 and analyzed for nuclear p65 subcellular localization. In untreated cells, p65 protein is primarily localized in the cytoplasm (Figure 5a1 & 5a2). As expected, TNFα treatment dramatically increased nuclear p65 translocation (Figure 5b1 & 5b2, 5d1 & 5d2), but BV6 treatment did not increase p65 nuclear translocation (Figure 5c1 & 5c2).

### Radiation activates the canonical p65/p50 and p50/p50 NF-κB

**Figure 6:**
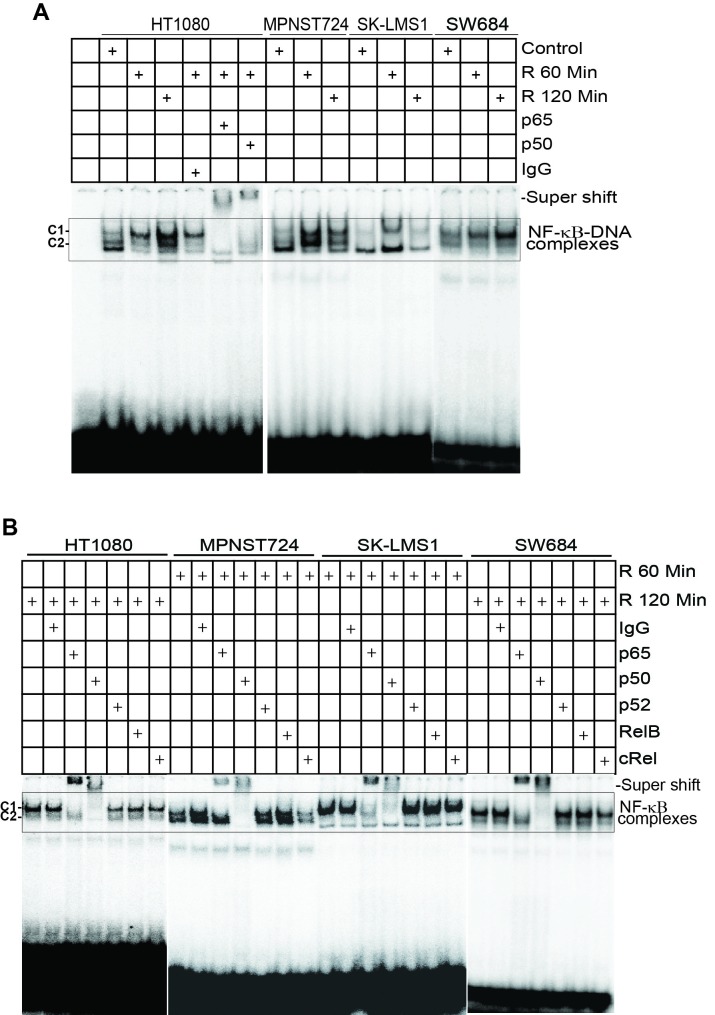
Radiation activates the canonical NF-κB **A.** Tumor cells were irradiated at a dose of 50 Gy and nuclear extracts were prepared at 1 and 2 h after culture. Nuclear extracts were then analyzed for canonical NF-κB activity using EMSA with NF-κB consensus sequence-containing DNA probe as in Figure 3. Anti-p65 and anti-p50 antibodies were used to identify the canonical NF-κB-DNA complexes. C1: p65/p50 heterodimer complex; C2: p50/p50 homodimer complex. **B.** The tumor cells were irradiated and cultured for 1 and 2h, respectively. Nuclear extracts were then analyzed for NF-κB activation using EMSA as in A. Antibodies that are specific for the five indicated NF-κB Rel subunits (p65, p50, p52, RelB, and cRel) were used to identify the NF-κB-DNA complexes.

All four human STS cells exhibit weak constitutive NF-κB activity (Figure 6A). However, radiation induces rapid NF-κB activation in all four cell lines within 60 minutes post radiation (Figure 6A). NF-κB subunit-specific antibody-based supershift assays indicate that no p52, RelB, or cRel DNA-binding activity was detectable in irradiated human sarcoma cells (Figure 6B). Two types of NF-κB-DNA complexes were detected. The C1 complex represents the canonical NF-κB p65/p50 heterodimer, whereas the C2 complex represents the NF-κB p50/p50 homodimer (Figure 6B). Therefore, we have determined that radiation activates both the canonical NF-κB p65/p50 heterodimer and the NF-κB p50/p50 homodimer.

### Radiation induces activation of canonical NF-κB that directly regulates TNFα expression to induce tumor cell death

**Figure 7:**
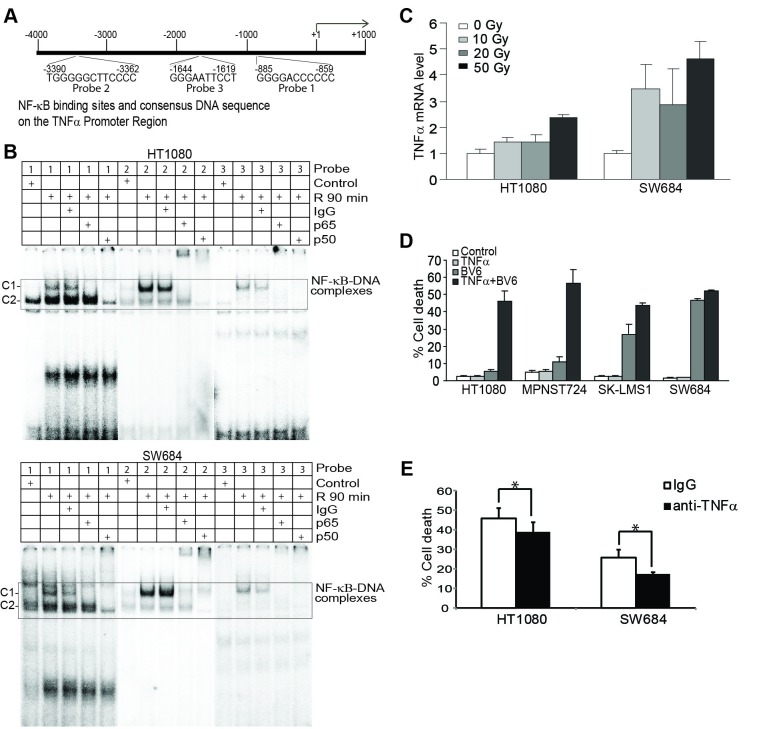
Radiation-induced NF-κB directly regulates TNFα transcription that acts in concert with BV6 to induce tumor cell death **A.** The human *TNFα* promoter structure. The three NF-κB-binding consensus sequence elements are indicated at the bottom of the bar. **B.** Tumor cells were irradiated at a dose of 50 Gy and then cultured for 90 min. Nuclear extracts were prepared and analyzed for NF-κB activation using EMSA with the three DNA probes of the human *TNFα* promoter as indicated in A. **C.** Tumor cells were irradiated with the indicated dose and cultured for 24h. Cells were then analyzed for human TNFα mRNA level using quantitative PCR. **D.** Tumor cells were treated as indicated for 24h and analyzed for cell death using PI staining and flow cytometry analysis. **E.** Tumor cells were irradiated and then cultured in the presence of BV6 (5 μM). IgG or anti-TNFα neutralization antibody (200 μg/ml) was added to the culture, respectively, for 24h. Cell death was analyzed by PI staining and flow cytometry analysis. *p < 0.05.

Analysis of the human *TNFα* promoter DNA sequence identified three potential NF-κB-binding DNA sequences (Figure 7A). Analysis of DNA-protein interactions revealed that radiation-induced canonical NF-κB binds to all three NF-κB binding sites in human STS cells (Figure 7B). In probes 1 and 2 regions, both p65/p50 and p50/p50 complexes were detected. In probe 3 region, only the p65/p50 complex was detected (Figure 7B). Consistent with NF-κB activation and binding to the TNFα promoter region, radiation induced an increase in TNFα mRNA level (Figure 7C).

### Radiation up-regulates TNFα expression to induce tumor cell death

Tumor-producing TNFα has been shown to induce tumor cell death in an autocrine manner [19, 21]. However, we observed that all four human sarcoma cell lines are not sensitive to TNFα-induced cell death (Figure 7D). A sublethal dose of BV6 significantly increased the sensitivity of human sarcoma cells to TNFα-induced cell death (Figure 7D). Next, tumor cells were irradiated and cultured in the presence of BV6 and a TNFα neutralization antibody. TNFα neutralization antibody significantly decreased radiation and BV6-induced cell death (Figure 7E).

### Radiation activates the canonical NF-κB to activate FAS transcription

**Figure 8:**
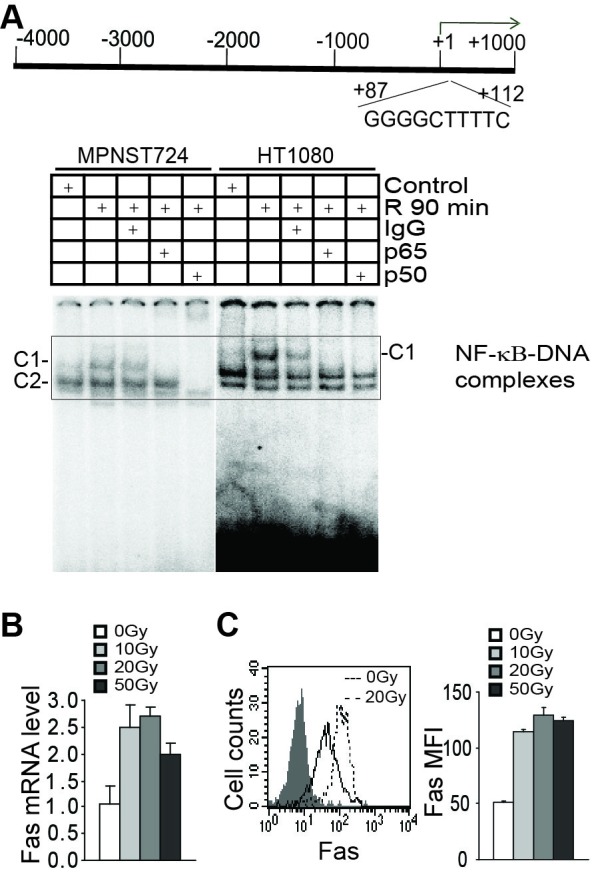
Radiation induces NF-κB to regulate FAS transcription **A.** The human *FAS* promoter structure. The NF-κB-binding consensus sequence element is indicated at the bottom of the bar. Tumor cells were irradiated at a dose of 50 Gy and then cultured for 90 min. Nuclear extracts were prepared and analyzed for NF-κB activation using EMSA with the DNA probe of the human *FAS* promoter as indicated in the top panel. **B.** HT1080 tumor cells were irradiated with the indicated doses and cultured for 24 hours. Cells were then analyzed for human Fas mRNA levels using quantitative PCR. **C.** HT1080 tumor cells were irradiated with the indicated doses for 24 hours. The cell surface Fas was stained with Fas-specific antibody and analyzed by flow cytometry. Representative plots are presented at the left. Fas MFI was quantified and presented at the right.

NF-κB is a known *FAS* transcription activator [46-48]. Analysis of the *FAS* promoter DNA sequences identified one potential NF-κB-binding consensus sequence (Figure 8A). EMSA analysis revealed that radiation-induced canonical NF-κB binding to this DNA element. Use of p65- and p50-specific antibodies indicates that NF-κB p65/p50 and p50/p50 complexes bind to the *FAS* promoter in MPNST724 cells, whereas only p65/p50 heterodimer binds to the *FAS* promoter in HT1080 cells (Figure 8A). Quantitative PCR analysis indicates that radiation increases Fas expression level (Figure 8B) and flow cytometry analysis reveals that radiation increases Fas protein level on the surface of tumor cells (Figure 8C).

### Radiation induces both apoptosis and necroptosis of tumor cells

**Figure 9:**
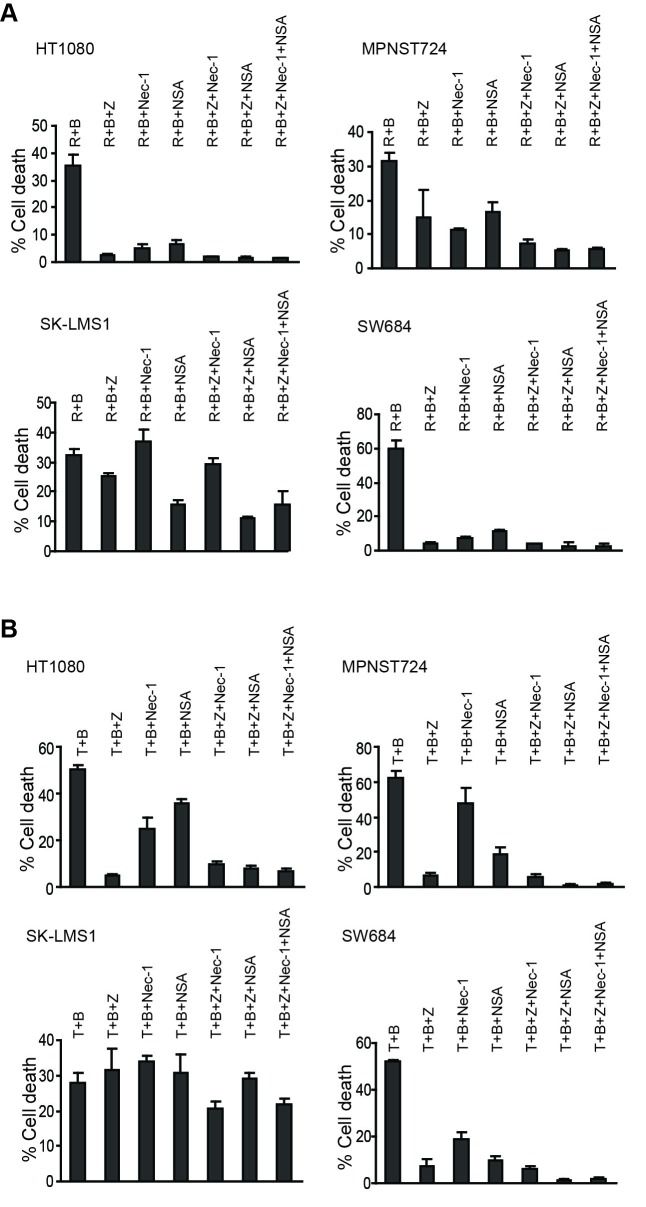
Radiation and TNFα induce apoptosis and necroptosis **A.** Tumor cells were irradiated (R) at a dose of 50 Gy and then cultured in the presence of BV6 (B, 5 μM) plus Z-VAD (Z, 10 μM), Nec-1 (10 μM), and NSA (2 μM), either alone or in combinations, for 24 hours. Cell death was determined by PI staining and flow cytometry analysis. Column: mean, bar: SD. **B.** Tumor cells were cultured in the presence of TNFα (T, 100 U/ml) and BV6 (B, 5 μM) plus Z-VAD (Z, 10 μM), Nec-1 (10 μM), and NSA (2 μM), either alone or in combinations, for 24h, and analyzed for cell death as in A. Column: mean; Bar: SD.

To test the relative contributions of apoptosis and necroptosis in radiation-induced cell death, we made use of the caspase inhibitor Z-VAD-fmk, an allosteric RIP1 kinase inhibitor Necrostain-1 (Nec-1), and the mixed linage kinase-like (MLKL) protein inhibitor Necrosulfonamide (NSA). Z-VAD effectively blocked radiation and BV6-induced cell death in three of the four human sarcoma cells. However, Nec-1 and NSA also inhibited radiation and BV6-induced cell death in the same three human sarcoma cell lines (Figure 9A). Similarly, Z-VAD blocked TNFα and BV6-induced cell death in three of the four STS cell lines. Nec-1 blocked cell death in HT1080 and SW684 cells, whereas NSA inhibited cell death in MPNST724 and SW684 cells (Figure 9B).

### BV6 modulates both the apoptosis and necroptosis pathways to increase TNFα-induced cell death

**Figure 10:**
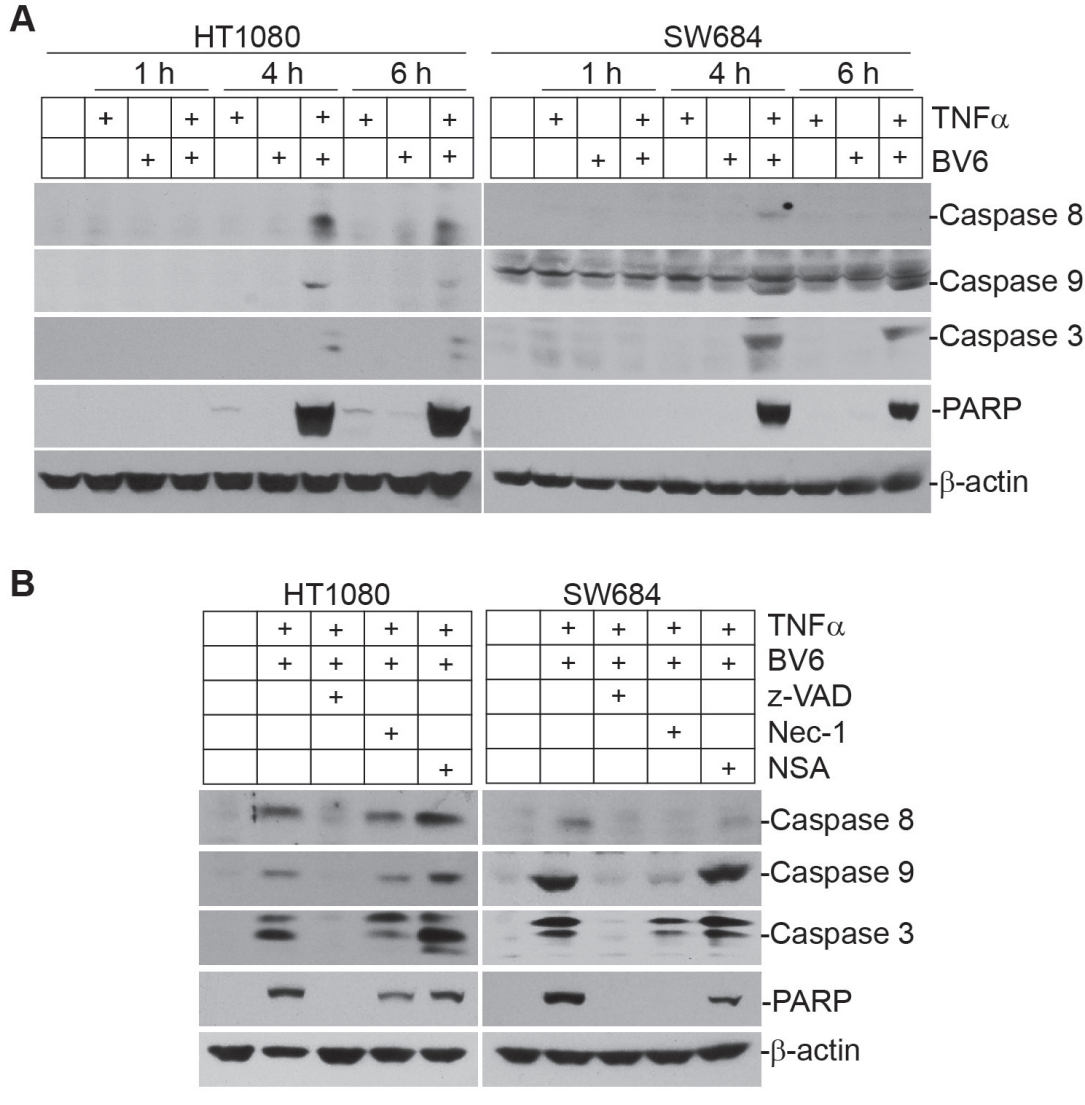
TNFα and BV6 cooperate to activate sequential necroptosis and apoptosis **A.** Tumor cells were cultured in the presence of TNFα (100 U/ml), BV6 (5 μM), or both for the indicated time and analyzed by Western blotting analysis for the indicated caspases. PARP was used as an apoptosis indicator. **B.** Tumor cells were cultured in the presence of TNFα (100 U/ml), BV6 (5 μM), plus Z-VAD (10 μM), Nec-1 (10 μM), or NSA (2 μM), respectively for 4 h, and analyzed by Western blotting for the indicated caspases and PARP.

Analysis of human tumor cells after BV6 treatment indicates that a sublethal dose of BV6 alone is insufficient to induce caspase activation (Figure 10A). TNFα alone also failed to induce caspase activation. However, a combined treatment with TNFα and BV6 activated caspases 8, 9, and 3, and induced PARP cleavage. To dissect the relative roles of apoptosis and necroptosis in tumor cell death, we treated tumor cells with BV6 and TNFα in the presence of apoptosis and necroptosis inhibitors. As expected, inhibition of apoptosis with Z-VAD blocked activation of caspases 8, 9, and 3, and blocked activation of apoptosis as indicated by lack of PARP cleavage (Figure 10B). Interestingly, Nec-1 also reduced caspase activation in both HT1080 and SW684 cells and decreased apoptosis as indicated by decreased PARP cleavage (Figure 10B). These observations suggest that BV6 and TNFα synergistically induce apoptosis and necroptosis that sequentially mediate tumor cell death.

### BV6 enhances radiation-induced xenograft tumor growth suppression *in vivo*

**Figure 11:**
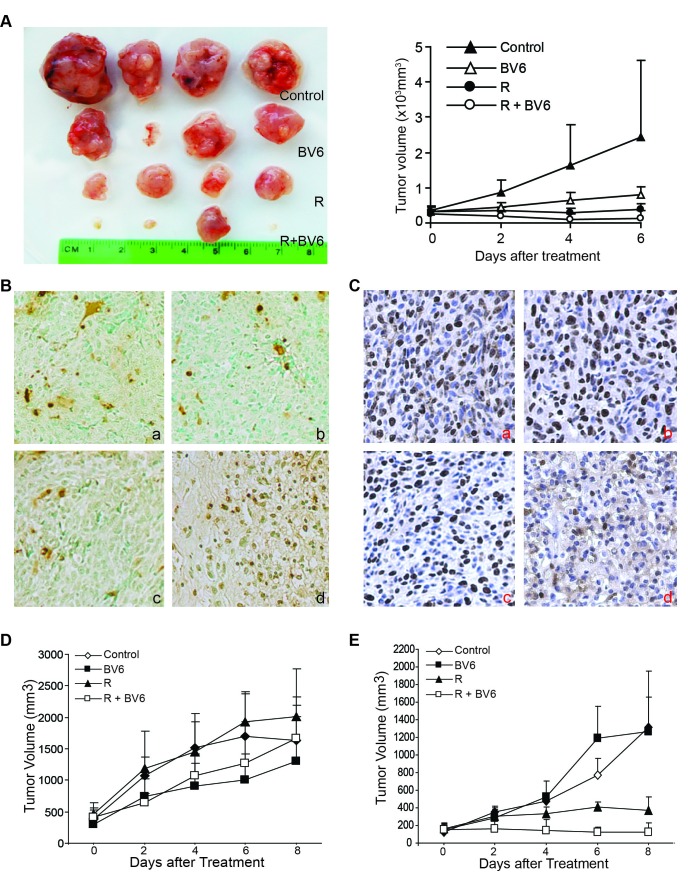
BV6 enhances radiation-mediated tumor growth suppression *in vivo* **A.** HT1080 cells (2.5×10^6^/mouse) were transplanted to athymic nude mice. Ten days after tumor injection, the established xenograft tumors were either untreated or irradiated using an external beam of radiation at a dose of 8 Gy (n=4 each treatment group). The mice were then either untreated or injected with BV6 i.v. (5 mg/kg body weight) every two days for four treatments. Tumors were dissected and presented at the left panel. The tumor xenograft growth kinetics are presented in the right panel. **B.** Xenograft tumor tissues, as shown in A, were dissected from mice and analyzed by immunohistochemical staining for *in situ* TUNEL. The brown color indicates apoptotic cell death. a: tumor from control mice, b: tumor from BV6-treated mice, c:tumor from radiated mice, and d: tumor from combined BV6 and radiation-treated mice. **C.** Xenograft tumor tissues, as shown in A, were dissected from mice and analyzed by immunohistochemical staining for Ki67 protein levels. The brown color indicates proliferating Ki67-positive tumor cells. a: tumor from control mice, b: tumor from BV6-treated mice, c:tumor from radiated mice, and d: tumor from combined BV6 and radiation-treated mice. **D.** Mouse sarcoma CMS4-met cells (2.5×10^5^/mouse) were transplanted to BALB/c mice. Ten days after tumor injection, the established tumors were either untreated or irradiated using an external beam of radiation at a dose of 8 Gy (n=4 each treatment group). The mice were then either untreated or injected with BV6 (5 mg/kg body weight) every two days for four treatments. The tumor growth was measured over time. Shown are the tumor growth kinetics. **E.** CMS4-met cells were injected to BALB/c mice and treated as in A except the radiation dose was increased to 20Gy. Shown are the tumor growth kinetics.

HT1080 cells were transplanted to nude mice. The tumor xenografts were then irradiated with a dose of 8 Gy, followed by BV6 treatment. The human sarcoma xenografts grew rapidly *in vivo* (Figure 11A). The post hoc pairwise comparisons showed that there were statistically significant differences between groups within days and between days within group. On day 4, the radiation group and the radiation+BV6 group had significantly lower mean tumor volume than the control group. On day 6, the radiation only, BV6 only, and radiation+BV6 had significantly lower mean tumor volumes than the control group, and the radiation+BV6 group had a significantly lower mean tumor volume than BV6 only (*p* < 0.01). Overall, combined radiation and BV6 eradicated three of the four tumors (Figure 11A).

Analysis of apoptotic cell death of the tumor tissues by an *in situ* TUNEL assay indicates that radiation increases apoptosis of tumor cells as compared to the control and BV6-treated tumors (Figure 11B). In the combined radiation and BV6-treated tumor tissues, almost all cells are apoptotic (Figure 11Bd). Analysis of tumor cell proliferation with Ki67 reveals that radiation also decreases the xenograft tumor cell proliferation, and combined radiation and BV6 treatment exhibited greater suppression of tumor cell proliferation than radiation and BV6 alone (Figure 11Cd).

### BV6 enhances radiation-induced syngeneic tumor growth suppression *in vivo*

Recent studies indicate that the host immune system plays a key role in radiation-mediated tumor suppression [1, 2, 4, 49, 50]. We then extend our study to an immune competent mouse tumor model. Mouse sarcoma CMS4-met cells were injected into syngeneic mice. The established tumors were irradiated with an external beam of radiation (8 Gy) and treated with BV6 as in the xenograft tumor model. Surprisingly, although both radiation and BV6 suppressed the xenograft tumor growth (Figure 11A), neither radiation nor BV6 suppressed the synergetic tumor growth in this immune competent syngeneic mouse tumor model (Figure 11D). Although a radiation dose of 8 Gy was commonly used in mouse tumor models [5], recent studies have shown that a higher dose of external beam radiation (20-40 Gy) is more effective at inducing an anti-tumor immune response [49]. We therefore increased the radiation dose to 20 Gy. One mouse in the radiation group and one mouse in the combined treatment group did not respond to radiotherapy due to a missed external beam of radiation of the tumor and therefore were excluded from the study. Analysis of tumor growth kinetics indicates that tumor volume was steadily increased across the measurement days in the control and BV6-treated mice. A slight increase in tumor volume was seen from day 0 to 2 with little to no change from day 2 to 8 in the radiation group. No change in tumor volume was seen across the measurement days in the combined radiation and BV6 group, indicating complete tumor suppression (Figure 11E). Within day 2 there were no significant differences between the 4 groups. Within day 4, the combined radiation and BV6 group had significantly lower tumor volume than the BV6 only group (*p* < 0.01). Within day 6, the combined radiation and BV6 group had significantly lower tumor volume than the BV6 only and control groups, and the radiation only group had significantly lower tumor volume than the BV6 only group. Within day 8, the combined radiation and BV6 group had significantly lower tumor volume than the BV6 only and control groups (*p* < 0.01).

### Radiation induces a Th1/Tc1 T cell response in the tumor microenvironment in the syngeneic tumor-bearing mice

**Figure 12:**
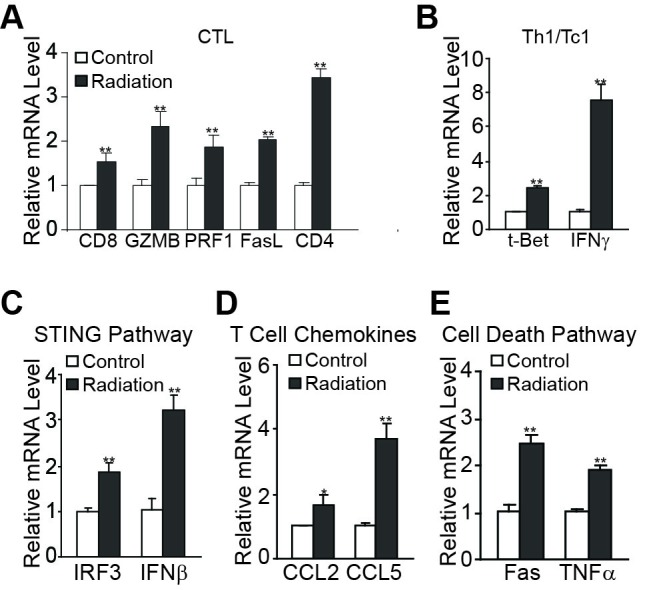
Radiation induces T cell immune responses in the tumor microenvironment Tumors of CMS4-met tumor-bearing mice were irradiated at a dose of 20Gy as shown in Figure 11E. Tumors were excised 8 days after radiation and RNA was isolated from the tumor tissues of untreated control and irradiated mice and analyzed by quantitative PCR for signature genes of T cells (**A.)**, Th1/Tc1 T cells (**B.)**, the STING pathway (**C.)**, T cell chemokines (**D.)**, and TNF cell death pathways (**E.)**. RNA from tumor tissues of three irradiated mice was pooled and RNA from tumor tissues of three untreated mice was pooled and used to prepare cDNA. The expression levels of genes in the control group were arbitrarily set to 1 for each subset of genes. The expression level of genes in the irradiated group is expressed as fold change over the control. *p<0.05, **p<0.01.

We next used quantitative PCR to analyze tumor tissues for the effects of radiation on the expression of signature genes for T cells and T cell linage-specific transcription factors. Tumors were excised and extracted for total RNA. Real-time RT-PCR analysis revealed that radiation induces an increase of CD4 and CD8 T cells (Figure 12A). Furthermore, the expression levels of IFNβ and t-Bet, the signature genes for Tc1/Th1 T cells, are also dramatically induced by radiation (Figure 12B). We also observed that radiation up-regulates perforin and granzyme B, two essential effectors of the perforin pathway. FasL is also up-regulated in the tumor microenvironment (Figure 12A). We next sought to determine whether radiation activates the STING pathway [49, 51] to activate T cells in the sarcoma tumor microenvironment. Quantitative PCR analysis revealed that IRF3 and IFNβ, two key mediators of the STING pathway, is up-regulated (Figure 12C). It has been reported that NF-κB is a transcriptional activator of the T cell chemokines CCL2 and CCL5 [52]. Analysis of the tumor tissues revealed that radiation up-regulates both CCL2 and CCL5 (Figure 12D). In addition, consistent with what was observed in irradiated tumor cell lines *in vitro*, Fas and TNFα are up-regulated by radiation in the tumor microenvironment (Figure 12E). Taken together, our data suggest that radiation not only activates the STING pathway to induce Th1/Tc1 T cell activation but also activates the canonical NF-κB to increase CCL2 and CCL5 to induce Th1/Tc1 T cell tumor infiltration.

## DISCUSSION

Radiation is known to activate NF-κB [34, 53-56], and NF-κB has been shown to confer tumor cell radioresistance [54]. However, NF-κB has also been shown to be required for radiation-induced cell death [34, 52]. In this study, we demonstrated that radiation rapidly induces activation of the canonical NF-κB. We further demonstrated that radiation activates both the p65/p50 heterodimer and p50/p50 homodimer of the canonical NF-κB that directly binds to the *TNFα* and *FAS* promoters, respectively, to activate their expression. NF-κB-activated TNFα then induces tumor cell death in an autocrine mechanism, whereas increased Fas in the tumor cells may increase tumor cell sensitivity to FasL-mediated cytotoxicity of tumor-reactive CTLs in the tumor microenvironment [57]. Therefore, we determine that radiation suppresses tumor cell growth at least in part through activation of the NF-κB-TNFα/Fas axis in the target tumor cells.

In agreement with early reports that BV6 induce cIAP1 and cIAP2 degradation to increase NIK stability to activate the alternate NF-κB [21, 58], we observed that BV6 induces the alternate NF-κB activation. However, the alternate NF-κB activation occurs at a late stage after BV6 treatment. The role of alternate NF-κB in BV6-mediated sarcoma and colon carcinoma cell death requires further study.

It is known that TNFα induces both apoptosis and necroptosis and these two cell death pathways often compensate for each other [59, 60]. Strikingly, we demonstrated that BV6 enhances TNFα function in the induction of tumor cell apoptosis and necroptosis. However, the BV6 and TNFα-induced apoptosis and necroptosis pathways do not compensate for each other since blocking either caspases alone or RIP1/RIP3 alone still inhibits cell death. It has also been shown that cIAP1 and cIAP2 use their E3 ligase activity to ubiquitylate RIP1 in order to mediate caspase 8 activation [61]. RIP1 is critical for caspase-8 activation-induced by Smac mimetic [61]. In this study, we observed that inhibition of RIP1 but not RIP3 diminishes caspase 8 activation, whereas inhibition of RIP1 or RIP3 both blocks radiation/TNFα-induced cell death. These results suggest that RIP1 is required for both apoptosis and necroptosis induced by radiation or TNFα and that RIP1 acts upstream of caspase 8.

The host immune system plays an essential role in radiation-mediated tumor suppression [1, 2, 4, 49, 50, 56]. In this study, we observed that radiation induced a dramatic increase in both CD4 and CD8 T cells in the tumor microenvironment. Furthermore, the tumor-infiltrating T cells are preferentially Tc1 and Th1 phenotypes as characterized by the increased expression of signature genes tBet and IFNβ. These tumor-infiltrating T cells are also activated by radiation since effector molecules such as perforin, granzyme B, and FasL are all high in the radiation-treated tumor than in untreated control tumors. It has recently been shown that antigen presenting cells take dsDNA fragments from irradiated tumor cells to activate the STING pathway in order to activate tumor-reactive CTLs [49, 51]. We observed here that radiation induces IRF3 and IFNβ, two key mediators of the STING pathway, in the tumor microenvironment. Therefore, radiation may activate the STING pathway to induce Tc1/Th1 T cell activation in the sarcoma tumor microenvironment.

**Figure 13:**
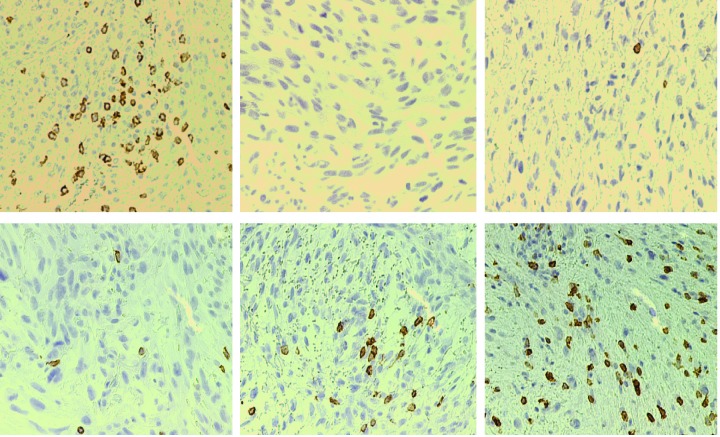
CD8^+^ cytotoxic T lymphocyte infiltration levels in human sarcoma Six Human fibrosarcoma tissues were stained with CD8-specific mAb. Brown color indicates tumor-infiltrating CD8^+^ CTLs. Brown color indicates CD8-specific staining, and the tissues were counterstained with hematoxylin. Each panel represents representative image of specimen from one patient.

In addition, it is possible that radiation-induced NF-κB activation in tumor cells might also be the link to radiation-induced T cell infiltration and activation in the tumor microenvironment [35]. It has recently been shown that NF-κB enhances the expression of T cell chemokines CCL2 and CCL5 to increase T cell tumor infiltration and tumor rejection in mouse tumor models and human lung cancer patients [52]. NF-κB activation in dying cells also determines cross-prming of CD8^+^ T cells [31]. We observed that radiation rapidly activates NF-κB in human sarcoma cells and increases both CD4^+^ and CD8^+^ T cells in the tumor microenvironment. Increased T cell tumor infiltration is correlated with increased CCL2 and CCL5 expression in the tumor tissues. It is thus likely that, in addition to the activation of the STING pathway to activate T cells, NF-κB may also up-regulate the T cell chemokines CCL2 and CCL5 in the tumor cells to attract T cell infiltration into the tumor microenvironment. Therefore, it is likely that radiation not only induces activation of the STING pathways to activate the Th1/Tc1 T cells but also up-regulates NF-κB to activate CCL2 and CCL5 to attract the activated Th1/Tc1 cells infiltration into the tumor microenvironment to suppress tumor development. CTL infiltration level in human sarcoma varies greatly from patients to patients. Analysis of tumor tissues from six human sarcoma patients indicates that half of the tumor tissues exhibit few to no CTL infiltration (Figure 13). Therefore, radiation-induced CTL infiltration may be a significant contributor for its tumor suppression efficacy.

**Figure 14:**
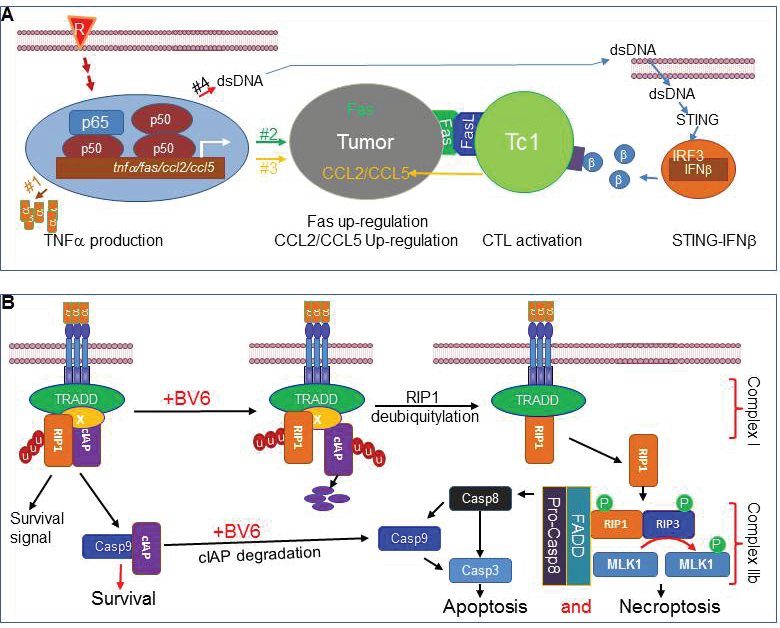
Model of radiation modulation of NF-κB and CTL anti-tumor immune response in the tumor microenvironment **A.** Radiation initiates at least four molecular effects: 1) radiation activates the canonical NF-κB p65/p50 and p50/p50 complexes that directly bind to the *TNFα* promoter to activate its transcription; 2) radiation-activated canonical NF-κB p65/p50 and p50/p50 complexes also directly bind to the *FAS* promoter to activate its transcription to increase tumor cell sensitivity to apoptosis induction by FasL of tumor-infiltrating Tc1 cells; 3) radiation-activated NF-κB also up-regulates the T cell chemokines CCL2 and CCL5 to attract T cell infiltration to the tumor microenvironment; and 4) radiation-generated dsDNA fragments activate the STING pathway, as characterized by increased IRF3 and IFNβ expression, to induce T cell activation. **B.** TNFα in the tumor microenvironment induces tumor apoptosis and necroptosis in an autocrine manner, and RIP1 is a key molecular switch that determines whether TNFα induces cell death or survival signaling pathways. cIAP1 and cIAP2 ubiquitylates RIP1 in the TNFR cytoplasmic complex (complex I) and the ubiquitylated RIP1 promotes cell survival by preventing the formation of the death complex IIb. BV6 induces cIAP1 and cIAP2 degradation. Degradation of cIAP1 and cIAP2 leads to activation of caspase 9. Degradation of cIAP1 and cIAP2 also leads to deubiquitylation of RIP1. Deubiquitylated RIP1 dissociates from the membrane-bound TNFR complex I to form cytosolic complex IIb that mediates both caspase-dependent apoptosis and RIP3-dependent necroptosis.

We propose that radiation initiates at least four bystander events (Figure 14): 1) radiation activates the canonical NF-κB p65/p50 and p50/p50 complexes to up-regulate TNFα expression. Increased TNFα in the tumor microenvironment induces tumor apoptosis and necroptosis in an autocrine manner; 2) radiation-activated canonical NF-κB p65/p50 and p50/p50 complexes activate Fas expression to increase tumor cells to apoptosis induction by FasL of tumor-infiltrating Tc1 cells [5, 57, 62]; 3) radiation-activated NF-κB also up-regulates CCL2 and CCL5 in tumor cells to attract activated T cell infiltration to the tumor microenvironment; and 4) radiation activates the STING pathway to induce T cell activation. On the other hand, RIP1 acts as molecular switch that determines whether TNFα induces cell death or survival. cIAP1/2 ubiquitylates RIP1 in the TNFR cytoplasmic complex (complex I) and the ubiquitylated RIP1 prevents the formation of the death complex IIb to promote cell survival [59, 63]. BV6 induces cIAP1 and cIAP2 degradation. Degradation of cIAP1 and cIAP2 leads to two events: first, caspase 9 activation; and second, deubiquitylation of RIP1. Deubiquitylated RIP1 dissociates from the membrane-bound TNFR complex I to form cytosolic complex IIb that mediates both caspase-dependent apoptosis and RIP3-dependent necroptosis. Thus, radiation-induced NF-κB functions as a key molecular link between tumor cells and immune cells in the tumor microenvironment for radiation-mediated tumor suppression.

## MATERIALS AND METHODS

### Mice and tumor cells

Athymic nude mice (*nu/nu*) were obtained from Taconic Farm Inc. BALB/c mice were obtained for Charles River Laboratory. All mouse studies were carried out according to an approved protocol by Georgia Regents University Institutional Animal Care and Use Committee. All human tumor cell lines used in this study were obtained from American Type Culture Collection (Manassas, VA). ATCC has characterized these cells by morphology, immunology, DNA fingerprint, and cytogenetics.

### Reagents

BV6 was provided by Genetech Inc. TNFα were obtained from R&D System. Z-VAD was obtained from ApexBio. Necrostatin-1 (Nec1) was obtained from Selleckchem. Necrosulfonamide (NSA) was obtained from Calbiochem.

### Radiation

Tumor cells were suspended in culture medium and irradiated with the MarK 1 Irradiator (Shepherd & Associates, San Fernando, CA). Tumors of tumor bearing mice were irradiated using the SARRP Irradiator System (Xstrahl Life Sciences Suwanee, GA). Mice were anesthetized under constant isoflurane/O_2_ flow and tumors were localized with the SARRP imaging system. Tumors were then irradiated with external beams of x-ray at the designated doses.

### Immunohistochemistry

Colon cancer tissue microarray (TMA) slides were provided by the National Cancer Institute (NCI)-sponsored cooperative human tissue network. The TMAs were designed by NCI statisticians for high statistical power for examination of associations of markers with tumor stage, clinical outcome, and other clinic-pathologic variables. TMA slides were stained with anti-cIAP antibody (R&D System), and counterstained with hematoxylin (Richard-Allan Scientific, Kalamazoo, MI) as previously described [64].

### Tumor cell death assay

For cell death analysis, cells were stained with Propidium Iodide and analyzed by flow cytometry as previously described [64].

### Western blotting analysis

Western blotting analysis was performed as previously described [65]. Anti-cleaved caspase 8 (Cat#: AF705, 0.5μg/ml) and anti-cIAP1 (Cat#: AF8181, 1:500) were obtained from R&D systems. Anti-caspase 3 (Cat#: 9661, 1:1000), anti-caspase 9 (Cat#: 9501, 1:500), anti-PARP (Cat#: 9541, 1:250), anti-xIAP (Cat#: 2042, 1:500), anti-p100/p52 (Cat#: 4882, 1:1000), anti-IκBα (Cat#: 4814, 1:2000), anti-pIκBα (Cat#: 2859, 1:1000) antibodies were obtained from Cell Signaling. Anti-cIAP2 (Cat#: NB110-57030, 1:250) was obtained from Novus. Anti-β-actin (Cat#: A5441, 1:5000) was obtained from Sigma (St Louis, MO).

### Electrophoresis mobility shift assay (EMSA) of NF-κB activation

NF-κB activation was analyzed using NF-κB probe (Santa Cruz Biotech) (Table S1), and probes with NF-κB consensus sequences of the TNFα and Fas promoter regions (Table S1) as previously described [66]. Briefly, the end-labeled probes were incubated with nuclear extracts for 20 min at room temperature. Anti-p65, p52, p50, RelB and cRel antibodies (Santa Cruz Biotech) were included to identify specific NF-κB-DNA complexes. DNA-protein complexes were separated by electrophoresis in 6% polyacrylamide gels and identified using a phosphoimage screen (Molecular Dynamics) and the images were acquired using a Strom 860 imager (Molecular Dynamics).

### Subcellular localization analysis

SW620 cells were cultured for 24 hours and were untreated, treated with BV6 (5μM), treated with TNFα (100 U/ml), or treated with BV6 and TNFα for one hour. Cells were fixed with 4% paraformaldehyde for 30 minutes, rinsed with PBS, blocked for one hour, and incubated with anti-p65 antibody (Santa Cruz) for one hour. The cells were then washed three times with PBS and incubated with a Cy5-conjugated anti-rabbit IgG for one hour. The cells were washed four times with PBS and followed by incubation with HOECHST for 5 minutes. The cells were analyzed by confocal microscopy.

### Gene expression analysis

Fresh tumor tissues were homogenized in Trizol (Life Technologies) to isolate total RNA. cDNA was synthesized from total RNA and used for analysis of gene expression using gene-specific primers (Table S1) in the StepOne Plus Real-Time PCR System (Applied Biosystems).

### Tumor tissue proliferation and apoptosis

Tumor tissues were embedded in paraffin and sections were stained with anti-Ki67 antibody (BD Biosciences), and the ApopTag^®^ Plus Peroxidase *in situ* Apoptosis Kit (Millipore, Billerica, MA), respectively according to the manufacturer's instructions.

### TNFα neutralization

Tumor cells were irradiated and then cultured in the presence of BV6 plus either IgG (20 μg/ml) or anti-TNFα mAb (Biolegend, 20 μg/ml) for 24h. Cell death was then measured by PI staining and flow cytometry.

### Statistical analysis

Statistical analysis was performed using SAS 9.4 and statistical significance was assessed using an alpha level of 0.05. A repeated measures mixed model was used to examine differences in tumor volume between treatment groups across days. Kaplan-Meier analysis was used to examine differences in time to recurrence and survival time by cIAP1 protein level. Student's *t* test was also used to compare differences between control and treatment groups. A *p*<0.05 was taken as statistically significant.

## SUPPLEMENTARY TABLE


