# Supplementary material for: NF-κB functions as a molecular link between tumor cells and Th1/Tc1 T cells in the tumor microenvironment to exert radiation-mediated tumor suppression
Source: Oncotarget. 2016 Mar 21;7(17):23395–415. doi: 10.18632/oncotarget.8246 (PMC5029635; doi:10.18632/oncotarget.8246)
Supplement: Supplementary file 1 [file oncotarget-07-23395-s001.pdf]

NF-κB functions as a molecular link between tumor cells and Th1/Tc1 T cells in the tumor microenvironment to exert radiation-mediated tumor suppression

Supplementary Material

Table S1. Oligo sequences

Oligo Name	Use	Forward	Reverse
hNF-κB WT	EMSA	5’- AGTTGAGGGGACTTTCCCAGGC-3’	5’-GCCTGGGAAAGTCCCCTCAACT-3’
hTNFαP NF-κB1	EMSA	5'-TCGAGTATGGGGACCCCCCCTTAACGA-3'	5'-TCGTTAAGGGGGGGTCCCCATACTCGA-3'
hTNFαP NF-κB2	EMSA	5'-CTAAGCCCTGGGGGCTTCCCCGGGCCCCA-3'	5'-TGGGGCCCGGGGAAGCCCCCAGGGCTTAG-3'
hTNFαP NF-κB3	EMSA	5'-GCCTGCCTAGGAATTCCCAGCCCAAA-3'	5'-TTTGGGCTGGGAATTCCTAGGCAGGC-3'
hTNFα	qPCR	5’-GGCAGGTTCTCTTCCTCTCACATAC-3’	5’-GCTTGTCACTCGGGGTTCG-3’
hFasP NF-κB	EMSA	5’-CCACCGGGGCTTTTCGTGAG-3’	5’-CTCACGAAAAGCCCCGGTGG-3’
hFas	qPCR	5’-ATTATCGTCCAAAAGTGTTAAT-3’	5’-TGCATGTTTTCTGTACTTCCTT-3’
mCD8	qPCR	5’-ACCTGGACATCAGAGCCCCTTG-3’	5’-AATCCTACGCTTTGCCCACC-3’
mGZMB	qPCR	5’-GCCCACAACATCAAAGAACAGG-3’	5’-CCAACCAGCCACATAGCACAC-3’
mPRF1	qPCR	5’-CCTATGGCACGCACTTTATCACG-3’	5’-TTCACTGGAGACGCTGGCTTGG-3’
mFasL	qPCR	5'-GTCAGTTTTTCCCTGTCCATCTTG-3'	5'-CCTAATCCCATTCCAACCAGAGC-3'
mCD4	qPCR	5’-CCTCAAGATACCCCAGGTCTCG-3’	5’-CAAGGAAACCCAGAAAGCCG-3’
mTbx21 (mt-Bet)	qPCR	5’-TGTTCCCATTCCTGTCCTTCAC-3’	5’-TGCTGCCTTCTGCCTTTCC-3’
mIFNγ	qPCR	5’-CCATCAGCAACAACATAAGCGTC-3’	5’-TCTCTTCCCCACCCCGAATCAGCAG-3’
mIRF3	qPCR	5'-CACGCTACACTCTGTGGTTCTGC-3'	5'-GCTGGCTGTTGGAGATGTGC-3'
mIFNβ	qPCR	5’-AAGAGTTACACTGCCTTTGCCATC-3’	5’-CACTGTCTGCTGGTGGAGTTCATC-3’
mCCL2	qPCR	5’-CCCAATGAGTAGGCTGGAGAGC-3’	5’-TGGTTGTGGAAAAGGTAGTGGATG-3’
mCCL5	qPCR	5’-AGGAACCGCCAAGTGTGTGC-3’	5’-CCGAGTGGGAGTAGGGGATTAC-3’
mFas	qPCR	5’-ATGCTGTGGATCTGGGCT-3’	5’-TCACTCCAGACATTGTCC-3’
mTNFα	qPCR	5’-GCTCTTCTGTCTACTGAACTTCGGG-3’	5’-ATCTGAGTGTGAGGGTCTGGGC-3’
